# Inferior Vena Cava Filter Placement during Pregnancy: An Adjuvant Option When Medical Therapy Fails

**DOI:** 10.1155/2013/821635

**Published:** 2013-05-27

**Authors:** Sara Valadares, Fátima Serrano, Rita Torres, Augusta Borges

**Affiliations:** ^1^Maternidade Dr. Alfredo da Costa, Obstetrics Department, Centro Hospitalar Lisboa Central, São Sebastião da Pedreira, 1069-089 Lisbon, Portugal; ^2^Obstetrics and Gynecology Department, Medical Science School, Lisbon Nova University, 1169-056 Lisbon, Portugal; ^3^Maternidade Dr. Alfredo da Costa, Internal Medicine Department, Centro Hospitalar Lisboa Central, São Sebastião da Pedreira, 1069-089 Lisbon, Portugal

## Abstract

The authors present a case of a 27-year-old multiparous woman, with multiple thrombophilia, whose pregnancy was complicated with deep venous thrombosis requiring placement of a vena cava filter. At 15th week of gestation, following an acute deep venous thrombosis of the right inferior limb, anticoagulant therapy with low-molecular-weight heparin (LMWH) was instituted without improvement in her clinical status. Subsequently, at 18 weeks of pregnancy, LMWH was switched to warfarin. At 30th week of gestation, the maintenance of high thrombotic risk was the premise for placement of an inferior vena cava filter for prophylaxis of pulmonary embolism during childbirth and postpartum. There were no complications and a vaginal delivery was accomplished at 37 weeks of gestation. Venal placement of inferior vena cava filters is an attractive option as prophylaxis for pulmonary embolism during pregnancy.

## 1. Introduction

Pregnancy and postpartum are periods of higher risk of thromboembolic events. Physiological changes in hemostasis and dynamics of venous return, genetics, and environmental factors contribute to this increased risk [1].

## 2. Case Presentation

We report the case of a 27-year-old multiparous woman, with multiple thrombophilia, whose pregnancy was complicated with deep venous thrombosis (DVT) requiring placement of a vena cava filter. 

She had a history of a previous DVT in the left leg at age 20, three months after beginning hormonal contraceptives. At that time a protein C deficit was diagnosed and she started anticoagulation with warfarin. 

At age 24, she was referred to our preconceptional clinic and a complete thrombophilia screening confirmed the protein C deficit (21%) and revealed the existence of other thrombophilia: heterozygosity for both factor V Leiden and prothrombin G20210A mutations and a positivity for lupus anticoagulant. She was overweight (body mass index of 28 kg/m^2^). There was no history of smoking habits or other personal and familiar thrombotic risk. 

During her first pregnancy warfarin was switched to prophylactic low-molecular-weight heparin (LMWH)—nadroparin calcium. Pregnancy passed uneventfully and at 39 weeks she had a normal delivery of a 3740 g healthy baby. Anticoagulation with warfarin was restarted in the puerperium.

Two years later she had her second planned pregnancy and anticoagulation was switched to LMWH in therapeutic doses. At 13 weeks of gestation, by self-initiative, she reduced LMWH dosage. Two weeks later she presented to our outpatient clinic with an acute deep venous thrombosis (DVP) of the right inferior limb. The Doppler study reported “extensive deep vein thrombosis from external iliac vein to the initial portion of the intramuscular veins of gastrocnemius muscle, a total occlusion of the superficial femoral vein, popliteal veins, and intramuscular veins of gastrocnemius muscle and a partial occlusion of the common femoral and external iliac veins. The magnus saphena had mild dilatation and was partially filled by thrombus.” LMWH was increased to therapeutic doses and additional prophylactic measures (limb elevation and stockings elastic restraint) were taken. The determination of Anti Xa levels (0.56 U/mL) were within therapeutic rage. Doppler study at 18th week of gestation revealed “total occlusion of the common femoral vein and femoropopliteal axis without recanalization and the presence of intraluminal thrombus at *Magna* saphena.” After discussing the obstetric risks, warfarin was started to achieve an INR between 2.5 and 3. Another Doppler study at 20th week of gestation showed “sequels of recent phlebothrombosis in right lower limb veins, with popliteal vein and superficial femoral vein partially occluded by a thrombus.” Due to the high risk of peripartum pulmonary embolism, an inferior vena cava filter (IVCF) placement was proposed. At 30 weeks of gestation an ICVF *Trapease* was placed percutaneously through the healthy contralateral inferior vena cava, under local anesthesia and fluoroscopic control, below the entry of renal veins. The total amount of radiation was 0.012 Gy and the effective dose for fetus was 1.44 mSv. *Visipaque*, a nonionic and water soluble contrast, was used. At 32 weeks warfarin was switched to therapeutic nadroparin calcium dosage until birth. Pregnancy proceeded without further complications. Labor was induced with misoprostol at 37 weeks of gestation and resulted in a vaginal delivery of a healthy 3600 g baby with a 9/10 Apgar score.

Currently, the patient is under anticoagulation with warfarin and had a definite family planning method (office hysteroscopic insertion of *Essure System*). Importance of screening family members for thrombophilia was stressed to patient's GP.

## 3. Discussion

Pregnancy is a critical period for thromboembolic events, both in terms of risk factors and therapeutic options [2–5]. The risk increases in pregnancy, particularly during third trimester and puerperium, due to physiological changes. Thromboembolism is the most common direct cause of maternal mortality in developed countries and it is estimated that one in every 2500 pregnancies may be complicated by DVT or PE, and [1]. Therefore it is essential to optimize the antithrombotic prophylactic care principally in women with additional thrombophilic factors. 

This case-report highlights several complex issues: first, the need for effective anticoagulation in high-risk patients, that is, frequently overwhelmed by the fear of anticoagulants administration during pregnancy, secondly, the fetal risks associated with the diagnostic and therapeutic procedures that were used.

In our patient, even with therapeutic anticoagulation levels, complete resolution of venous obstruction was not achieved with LMWH, so dicumarol therapy was initiated despite our knowledge of its potential teratogenic and fetal anticoagulation effects. Coumarinic oral anticoagulants are classified as risk class X by FDA [6] and its use should be restricted to selected situations. The literature classically describes malformations in about 15 to 25% of fetuses exposed to warfarin treatment during first trimester; the period of the highest susceptibility is between the 6th and 9th week of gestation [7, 8]. However, recent studies showed that this risk was overestimated. A multicentric study with 666 pregnant women exposed to warfarin reveled a 0.6% incidence of fetal malformations [9]. 

Recurrent thromboembolism that occurs despite adequate anticoagulation is a condition where vena cava filters placement is indicated during a higher-risk period like peripartum. Although there are limited data available, vena cava filters have been used in the perinatal period, in patients with acute deep vein thrombosis [10]. In this context, in our patient, placement of an IVCF became an appealing option for preventing PE. There are two key aspects in IVCF placement. The first one is its location, usually below renal veins avoiding disruption of renal flow; however, in pregnant women, some authors place them above renal veins because of displacement risk related to extrinsic pressure caused by the pregnant uterus [11]. The second aspect concerns placement of a permanent filter in a young woman. Although long-term effects of permanent filters in young patients are not established, there is data reporting security of this procedure for general population, with a 20-year-followup period [12]. Nowadays a new generation of filters allows either permanent placement or its removal, when possible, once the thromboembolic risk disappears. This last type of filters has been proposed as more secure in pregnant women with high risk of PE during peripartum period; its placement during pregnancy and subsequent removal were demonstrated to be safe in small retrospective studies [10, 13–15]. In our case, attending to the irreversible risk factors (protein C deficit, heterozygosity for both factor V Leiden and Prothrombin G20210A and positive lupus anticoagulant) presented in our patient, we chose a permanent filter. 

The insertion was done under fluoroscopic control, the total dosage of radiation used was 8.33 times lower than 0.1 Gy—the dose required to produce biological effects on the fetus. Similarly, the effective dose to the fetus was 3.47 times less than the legal limit (5mSv—Directive 96/29/EURATOM) [16]. Radiation effects in embryo or fetus are multiple: lethality and teratogenesis or carcinogenesis and genetic mutations. In the first case the risk is zero at exposures below 0.1 Gy or 5mSv (most diagnostic tests for conventional Rx implies a lower exposure to 1mSv) [17]. If exposure occurs before implantation, “all-or-nothing” law is applied, resulting in miscarriage or in a safe fetus. In the second case there is little information available in the literature; there appears to be a slight risk of childhood leukemia after exposure *in utero* [18, 19]. *Visipaque*, the contrast medium used, has showed no teratogenic or fetal toxic effect in animal's studies [20].

ICVFs, especially temporary filters during peripartum period, are a good solution for pregnant women with increased thromboembolic risk factors, preventing pulmonary embolism without the hemorrhagic risks associated with anticoagulation therapy. Growing evidence demonstrates the safety of these procedures in younger women and pregnancy.
